# Short-term succinic acid treatment mitigates cerebellar mitochondrial OXPHOS dysfunction, neurodegeneration and ataxia in a Purkinje-specific spinocerebellar ataxia type 1 (SCA1) mouse model

**DOI:** 10.1371/journal.pone.0188425

**Published:** 2017-12-06

**Authors:** Austin Ferro, Emily Carbone, Jenny Zhang, Evan Marzouk, Monica Villegas, Asher Siegel, Donna Nguyen, Thomas Possidente, Jessilyn Hartman, Kailen Polley, Melissa A. Ingram, Georgia Berry, Thomas H. Reynolds, Bernard Possidente, Kimberley Frederick, Stephen Ives, Sarita Lagalwar

**Affiliations:** 1 Neuroscience Program, Skidmore College, Saratoga Springs, New York, United States of America; 2 Chemistry Department, Skidmore College, Saratoga Springs, New York, United States of America; 3 Institute for Translational Neuroscience, Department of Lab Medicine and Pathology, University of Minnesota, Minneapolis, Minnesota, United States of America; 4 Health and Exercise Science Department, Skidmore College, Saratoga Springs, New York, United States of America; 5 Biology Department, Skidmore College, Saratoga Springs, New York, United States of America; University of Nottingham, UNITED KINGDOM

## Abstract

Mitochondrial dysfunction plays a significant role in neurodegenerative disease including ataxias and other movement disorders, particularly those marked by progressive degeneration in the cerebellum. In this study, we investigate the role of mitochondrial oxidative phosphorylation (OXPHOS) deficits in cerebellar tissue of a Purkinje cell-driven spinocerebellar ataxia type 1 (SCA1) mouse. Using RNA sequencing transcriptomics, OXPHOS complex assembly analysis and oxygen consumption assays, we report that in the presence of mutant polyglutamine-expanded ataxin-1, SCA1 mice display deficits in cerebellar OXPHOS complex I (NADH-coenzyme Q oxidoreductase). Complex I genes are upregulated at the time of symptom onset and upregulation persists into late stage disease; yet, functional assembly of complex I macromolecules are diminished and oxygen respiration through complex I is reduced. Acute treatment of postsymptomatic SCA1 mice with succinic acid, a complex II (succinate dehydrogenase) electron donor to bypass complex I dysfunction, ameliorated cerebellar OXPHOS dysfunction, reduced cerebellar pathology and improved motor behavior. Thus, exploration of mitochondrial dysfunction and its role in neurodegenerative ataxias, and warrants further investigation.

## Introduction

Spinocerebellar ataxia type 1 (SCA1) is a progressive, autosomal dominant neurodegenerative disease caused by a CAG repeat expansion in the *ataxin-1* (*ATXN1*, *SCA1*) gene that produces a polyglutamine expansion in the coded protein. Pathogenic alleles house 39–82 glutamine and glutamine tract-length indirectly correlates with age of onset [1] likely due to strengthened stability [2–4] of expanded ATXN1 protein and altered function in its role as a regulator of gene expression and transcript splicing [5–7](reviewed in [2]). The primary site of SCA1 pathogenesis are cerebellar Purkinje neurons, the sole known cell type in which ATXN1 expression is both cytoplasmic and nuclear [8–11].

With their extensively branched dendritic arbors and long axons, Purkinje neurons require high metabolic activity [12, 13]. Mitochondrial generated ATP via oxidative phosphorylation (OXPHOS) is essential for synaptic trafficking of specialized proteins and vesicles to proximal and far distal regions as well as replenishment of the resting membrane potential via ion pumps, among many other functions [13]. Reliance of Purkinje neurons on OXPHOS activity is apparent in disorders of global mitochondrial dysfunction [14–18] and OXPHOS complex-associated disorders [14, 19], in which Purkinje neurons are critically vulnerable.

OXPHOS complex dysfunction has been reported in conjunction with misfolded protein-mediated pathogenesis in neurodegenerative disorders including early and late-stage Alzheimer’s disease [20], Parkinson’s disease [21, 22], and multiple system atrophy [22]. More recently, OXPHOS dysfunction has been identified within Purkinje neurons of a knock-in ATXN1[154Q] SCA1 mouse model [23]; where treatment with an antioxidant ameliorated the OXPHOS deficits [23].

In this study, we characterize OXPHOS dysfunction in a transgenic Purkinje-cell specific model of SCA1, the B05 mouse. B05 mice selectively overexpress the physiologically relevant ATXN1 mutant, ATNX1[82Q], in Purkinje neurons [24]. The mice develop an ataxic phenotype at 12 weeks of age, accompanied by accumulation of nuclear ATXN1[82Q] aggregates and eventual Purkinje degeneration [24]. Pathogenesis does not spread outside of the cerebellum; brainstem and spinal cord nuclei are spared, and mice are otherwise healthy. This model allows us to determine the consequence of Purkinje cell expression of polyQ expanded-ATXN1 on cerebellar OXPHOS in a transgenic model of SCA1.

We report here that OXPHOS complex I deficits are detectable in cerebellar preparations of B05 mice using genetic, biochemical and physiological means. Specifically, adult B05 cerebellum display upregulation of complex I genes, disassembly of complex I macromolecules and reduced complex I respiration. Moreover, we hypothesized that bypassing complex I via exogenous treatment of the complex II electron donor, succinic acid [25–27], may have therapeutic effects in the B05 mouse through the restoration of OXPHOS. Succinic acid, a Krebs cycle metabolite, oxidizes to fumarate via complex II, succinate dehydrogenase [28–30]. In the present study, we confirm that succinic acid in the cage drinking water elevates endogenous cerebellar succinate levels. Furthermore, treatment of symptomatic adult B05 mice for four weeks improves cerebellar OXPHOS respiration, prevents Purkinje cell atrophy and mitigates ataxia.

These findings suggest augmenting mitochondrial activity, through succinic acid treatment, may assist vulnerable neurons in defending against the prolonged internal assault of abnormally stabilized aggregation-prone proteins.

## Materials and methods

### Mice and succinic acid treatment

SCA1 B05 and A02 transgenic mice on an FVB/nJ background strain were gifts from Dr. Harry Orr (University of Minnesota). B05^+/-^ mice express the *ATXN1[82Q]* transgene and A02^+/-^ mice express the *ATXN1[30Q]* transgene, both under the control of the Purkinge cell-specific promoter *Pcp2/L7* [24, 31]. Wild type FVB/nJ mice were purchased from Jackson Laboratory (Bar Harbor, ME, 001800). Transgenic lines were bred to homozygosity (B05^+/+^ and A02^+/+^, referred to here as B05 and A02 for simplicity) and maintained along with wild type mice at the Skidmore College mouse facility in strict accordance with the recommendations established for the care and use of laboratory animals by the National Institute of Health and approved by the Skidmore College Institutional Care and Use Committee (IACUC) (*NIH Publications No*. *8023*, *revised 1978*). Transgene integration was verified in each generation by PCR. Mice were fed chow *ad libitum*. Cage drinking water was replaced with 0.75 mg/mL succinic acid in treated mice and administered *ad libitum* beginning at age four months. Water and treatment intake was measured daily. Cohort sizes of 8–14 mice per genotype were used for neuropathology, behavioral assays, metabolic assays and circadian rhythm assays. Cohort sizes of 3–4 mice per genotype were used for RNAseq, complex assembly analysis and oxygen respiration. Mice were euthanized by displacement of the cage air with 10–30% compressed carbon dioxide gas per minute. All animal protocols in this study were approved by the Skidmore College IACUC.

### RNA sequencing (RNA-Seq)

Gene expression profiling was conducted as recently published [32] on B05^+/-^ and wild type FVB cerebellar RNA at 5, 12 and 28 weeks of age. The chosen ages represent mild, moderate and severe ataxia [33] in heterozygotes. Three biological replicates were run per genotype. For the purpose of this study, statistically significant (P < 0.05) upregulation and downregulation of nuclear OXPHOS complex genes in B05^+/-^ compared to FVB were analyzed from the data set provided by the Orr lab (University of Minnesota).

### OXPHOS complex assembly western blot

To detect OXPHOS complex assembly, wild type untreated, B05 untreated and B05 treated whole cerebellar homogenates were prepared in lysis buffer [0.25 M Tris-HCl, pH 7.5 containing 1X protease inhibitors (Roche Biochemicals, Indianapolis, IN) and phosphatase inhibitor cocktails 2 and 3 (Sigma, St. Louis, MO)]. Samples were combined with 1X Laemmli reducing buffer at room temperature. 20 μg of unboiled samples were resolved in triplicate by SDS-PAGE, transferred to nitrocellulose membranes and blotted with an assembly-dependent total OXPHOS rodent antibody cocktail (AbCam ab110413, Cambridge MA). Bands were visualized on a C-DiGit Western Blot Scanner (Licor Biosciences, Lincoln, NE) and quantified using Image Studio densitometry software (Licor). CI, CII, CIII and CV band densities were averaged and normalized to the average CIV band density. Data is expressed as normalized means ± SEM.

### Mitochondrial respiration

Mitochondrial respiration experiments were adapted from Kuznetsov, et al. [34]; our methodology for this study is described elsewhere [35]. Briefly, whole cerebella from treated and untreated FVB and B05 mice were quickly extracted from CO_2_-asphyxiated wild type and B05 mice (treated and untreated) under IACUC guidelines and prepared in homogenate buffer (0.25 M sucrose, 0.5 mM EDTA, 50 mM Tris-HCl, pH 7.4), and added to the calibrated respirometer set to 30°C (Oxytherm System, Hansatech Instruments, UK). Tissue plasma membranes were permeabilized in the chamber with digitonin (15 μg/mL). Oxygen consumption was recorded in response to the sequential addition of glutamate/malate (Complex I substrate, 10 mM and 5 mM, respectively), ADP (1 mM), rotenone (Complex I inhibitor, 0.5 μM), succinic acid (Complex II substrate, 10 mM), antimycin A (Complex III inhibitor, 5 μM), TMPD/ascorbate (Electron donor, 0.5 mM and 2 mM, respectively) and cytochrome C (10 μM). Percent of total respiration following activator and inhibitor addition was calculated by normalizing the average oxygen consumption values by cerebellar wet weight and comparing it to the corresponding normalized intra-assay response to TMPD/ascorbate. Three biological replicates per genotype and treatment were averaged ± SEM.

### HPLC

HPLC respiration experiments were adapted from Ergönül and Nergiz [36]; our adapted methodology for this study is described in detail elsewhere [35]. In brief, PBS-washed cerebellar, cortical, liver and kidney tissue from treated and untreated FVB and B05 mice were finely minced in a chilled dounce homogenizer in 75:25 water: methanol, by volume. Following centrifugation at 1300 x g at 4°C for 30 minutes, supernatants were filtered through regenerated cellulose with a 10 kDa nominal molecular weight limit. Samples and known succinic acid standards were diluted in degassed 1 mM acetate buffer (pH = 5) mobile phase and run by HPLC through a Shodex KC-811 ion exclusion chromatography column with a length 30 cm and a particle size of 7 μm. Mobile phase velocity was set to 0.8 mL/minute and a UV-vis detector was set to 224 nm. Peak areas of standards were used to obtain a standard calibration curve. Succinic acid concentrations of samples were determined from the calibration curve, multiplied by the sample volume and divided by the original sample mass. All standards and samples were analyzed in triplicate.

### Neuropathology

Whole cerebellar tissue was quickly harvested from CO_2_-asphyxiated FVB and B05 mice (treated and untreated) under IACUC guidelines, and immediately fixed in ice cold 4% paraformaldehyde overnight. Following fixation, tissue was sunk in 30% cold sucrose over three days, embedded in O.C.T. Compound (Fisher Healthcare) and cut into 50 μm sagittal sections on a sledge microtome (ERMA ESM-100L, Tokyo, Japan). Following an established protocol [37], epitopes were unmasked by boiling three times for 15 seconds each in 0.01 M urea, blocked for 1 hour in 2% normal donkey serum and 0.3% Triton X-100 in 1X PBS, and incubated for 48 hours at 4°C in blocking solution containing goat calbindin antibody (SC-7691, Santa Cruz) at 1:500. Sections were washed four times in 1X PBS, and incubated for 48 hours at 4°C in blocking solution containing 1:500 Alexa 488-donkey anti-goat (ThermoFisher Scientific). Sections were washed and mounted onto microscope slides with glycerol-gelatin containing 4 mg/mL n-propyl gallate (Sigma) and imaged on an Olympus Fluoview 1200 upright microscope as previously described [38].

Confocal images of cerebellar primary fissures were captured from 20 μm z-stacks at 20X objective. Molecular layer thickness was measured in Image J by scorers that were blinded to the genotype and treatment conditions. Two designated 200 μm stretches of primary fissure along the Purkinje layer were identified in each image, and three random measurements were made within each 200 μm stretch from the proximal end of the Purkinje dendritic arbor at the base of the soma to the distal end of the dendritic arbor [35, 37]. Six measurements from three cerebellar sections per animal were averaged and expressed as the mean ± SEM.

To analyze Purkinje cell counts, cerebellar sections from A02 transgenic mice and B05 transgenic mice (treated and untreated) were prepared as described above, incubated in 11NQ anti-ATXN1 antibody (1:2000) [9], and detected with 1:500 Alexa 594-goat anti-rabbit secondary antibody (ThermoFisher Scientific). Following confocal imaging of primary fissures, two designated 200 μm stretches along the Purkinje layer were identified, and the number of ATXN1-positive nuclei within each stretch were counted manually [35] by blinded scorers. Six measurements from three cerebellar sections per animal were averaged and expressed as the mean number of soma ± SEM.

### Footprint testing

Gait assessment was measured by labeling hind feet of mice with blue or red non-toxic water-based paint, placing subjects on a paper-lined runway and allowing them to walk towards a goal box, as described in detail previously [33, 35]. Successful trials were defined as a minimum of five sequential footprints in which the subject walked towards the goal box without turning or stopping [35]. Step length, gait width, alternation coefficient, and linear movement were assessed from the footprints as described previously [33] by blinded scorers. Measurements from subjects within the same genotype and treatment cohorts were average and expressed ± SEM.

### Balance beam testing

Motor deficits and ataxia were assessed by the balance beam test as described previously [33, 35]. Specifically, mice were tested on a trial beam (12mm square) for 3 trials per day for three days. On the subsequent testing day, mice were run across 6 beams which alternated between square and round, and ranged from 28 mm to 6 mm, sequentially declining in thickness and radius. Mice were assayed on testing day (day 4) for the number of footslips made by their left hindfoot as they crossed beam three successfully, and by the number of successful attempts that it took for them to cross each beam. Videotaped footage was evaluated by blinded scorers to score the number of footslips and successful trials. Measurements from subjects within the same genotype and treatment cohorts were average and expressed ± SEM.

### Accelerating rotarod testing

Ataxia was further assessed by the accelerating rotarod protocol in which rod speed was gradually accelerated between 4 and 40 rpm over a 5-minute period, then maintained at a constant acceleration for up to five additional minutes [33, 35] (Rotallion Rotarod; PPP&G, Saint Paul, MN). Latency to fall was recorded over four trials per day for four consecutive days. Measurements from subjects within the same genotype and treatment cohorts were average and expressed ± SEM.

### Energy expenditure

Energy expenditure was assessed in A02 mice using metabolic cages (OxyMax, Columbus Instruments, Columbus, OH) equipped with O_2_ and CO_2_ analyzers according to the manufacturer’s instructions [39]. Following an 8-hour acclimatization period, energy expenditure was assessed continuously during a 24-hour dark-light period with *ad libitum* access to food and treated (eight females and seven males, age 300 +/- 16 days) or un-treated water (five females and eight males, age 297 +/- 14 days). The volume of oxygen consumed (VO_2_), volume of carbon dioxide produced (VCO_2_), energy expenditure (HEAT) and the respiratory exchange ratio (RER), the ratio of CO_2_ to O_2_, were recorded over time. Mean data points were plotted over time and t-tests were used to determine significant differences of each measure between treated and untreated male or female cohorts.

### Circadian rhythm analysis

Circadian rhythms were analyzed in mice as previously described for rats [40]. Briefly, A02 mice were housed in individual plastic cages equipped with a Nalgene running wheel (Mini Mitter, Bend, OR) and with *ad libitum* access to food and treated (eight females and seven males, age 300 +/- 16 days) or un-treated water (five females and eight males, age 297 +/- 14 days). Running wheel activity was assayed for ten days under a 12:12 light dark cycle (12:12LD) after four days of acclimation to the running wheel cages, followed by ten days in constant dim red light (DD). Monochromatic red light was produced using Kodak filter number one, and measured less than one lux at the top of each cage. Running wheel activity data was collected in 10-minute bins using VitalView software (Mini Mitter) and analyzed using ActiView software (Mini Mitter). Mean activity level was assayed in 12:12LD (XLD), the light phase of the LD cycle (XL), the dark phase of the LD cycle (XD) and in constant dark (XDD). Peak time of activity in 12:12LD (PHLD) and the free-running circadian period in DD (TAUDD) were also analyzed. To prevent circadian disruptions in the analysis, routine procedures occurred at random times during the light period of the LD cycle.

### Statistics

Statistical analyses are indicated in figure legends. In general, comparisons of statistical significance of difference by a single effect were evaluated by t-test. Comparisons of statistical significance of difference by multiple effects were evaluated by one- or two-way ANOVA and the Tukey post hoc test. T-tests and ANOVAs were conducted in GraphPad Prism. Running wheel cage data was analyzed in SAS using the GLM procedure. Data, where indicated, are represented as mean ± SEM.

## Results and discussion

### Oxidative phosphorylation complexes are dysfunctional in B05 mice cerebellum

Oxidative phosphorylation complex dysfunction was analyzed in B05 mouse cerebellum via three different measures. First, RNAseq analysis was performed on B05 and wild type cerebella prior to behavioral and neurological deficits of SCA1 (5 weeks of age) [24, 41], early stage SCA1 (12 weeks) and advanced stage SCA1 (28 weeks) [24, 32](Table 1). OXPHOS complex genes that were significantly (P< 0.01) downregulated (top) or upregulated (bottom) in B05 compared to age-matched wild type are noted in Table 1. The complex 4 gene, Cox6b2, which is highly expressed in Purkinje cells [42] and the ATPase gene, Lhpp, were downregulated in B05 cerebellum compared to control cerebellum at 5 weeks of age. Both genes remained downregulated at 12 weeks of age. In contrast, no OXPHOS genes were upregulated in B05 at 5 weeks. At 12 weeks of age, the complex I genes Ndufb2 and Ndufb3, complex II genes Sdhc and Sdhd, complex III gene Uqcrh, complex 4 genes Cox5a, Cox6a1, and Cox4i1 and ATPase gene Atp5g3 were downregulated. At 28 weeks of age, OXPHOS downregulation was not detected. Upregulation of complex I genes Ndufa11, Ndufb5, Ndufs5 and Ndufv2, complex III gene Uqcrq, complex IV genes Cox7a21 and Cox7b and ATPase gene Atp5j2 were upregulated at 12 weeks. Upregulated expression of Ndufa11, Uqcrq, Cox7a21 and Atp5j2 persisted through 28 weeks. Additionally, at 28 weeks, complex I genes Ndufa11, Ndufa6, Ndufa7, Ndufb10, Ndufb7, Ndufb8, Ndufb9 and Ndufs8, complex II gene Sdhc, and complex III gene Uqcr11 were upregulated. The greatest downward fold change in B05 compared to wild type was in Cox6b2 (log 2-fold change of 1.77038 and 1.27923 at 5 weeks and 12 weeks, respectively) and the greatest upward fold change was in Ndufs5 (log 2 fold change of -1.67463).

**Table 1:** RNAseq analysis of OXPHOS complex genes in B05 cerebellar tissue versus wild type tissue.

	**Downregulation of OXPHOS complex genes in B05** **(log 2 fold change)**				
	*Complex I*	*Complex II*	*Complex III*	*Complex IV*	*ATPase*
*vs Wt-5 wks*				*Cox6b2 (1.77038)	Lhpp (0.641673)
*vs Wt-12 wks*	Ndufb2 (0.634449)	Sdhc (0.515247)	Uqcrh (0.335416)	Cox5a (0.404022)	Atp5g3 (0.342982)
	Ndufb3 (0.457995)	Sdhd (0.477251)		Cox6a1 (0.514417)	Lhpp (0.780756)
				*Cox6b2 (1.27923)	
				Cox4i1 (0.368788)	
*vs Wt-28 wks*					
	**Upregulation of OXPHOS complex genes in B05** **(log 2 fold change)**				
	*Complex I*	*Complex II*	*Complex III*	*Complex IV*	*ATPase*
*vs Wt-5 wks*					
*vs Wt-12 wks*	Ndufa11 (-0.349446)		Uqcrq (-0.416653)	Cox7a2l (-0.328557)	Atp5j2 (-0.516911)
	Ndufb5 (-0.356859)			Cox7b (-0.326646)	
	Ndufs5 (-1.67463)				
	Ndufv2 (-0.552158)				
*vs Wt-28 wks*	Ndufa11 (-0.509067)	Sdhc (-0.498714)	Uqcr11 (-0.454752)	Cox7a2l (-0.583099)	Atp5j2 (-0.493386)
	Ndufa6 (-0.507489)		Uqcrq (-0.650516)		
	Ndufa7 (-0.480316)				
	Ndufb10 (-0.4287)				
	Ndufb7 (-0.502352)				
	Ndufb8 (-0.599094)				
	Ndufb9 (-0.434831)				
	Ndufs8 (-0.450487)				

Top table lists genes upregulated (P<0.01) in B05 compared to wild type at 5, 12 and 28 weeks. Bottom table lists genes downregulated (P<0.01) in B05 compared to wild type at 12 and 28 weeks. Complex genes were not downregulated at 5 weeks.

*Indicates Purkinje-cell enriched genes [42].

**Fig 1:**
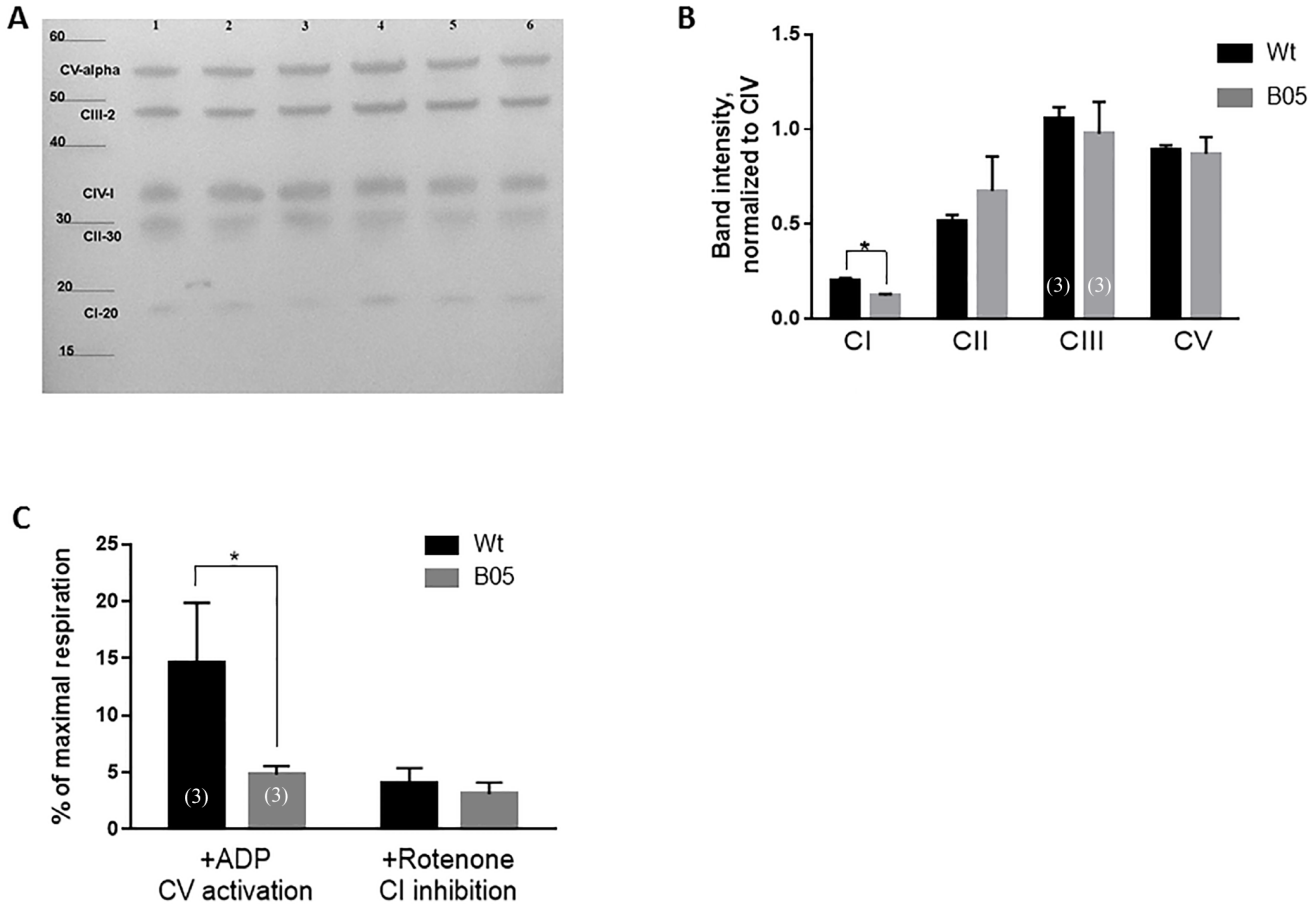
OXPHOS complex I dysfunction in adult B05 cerebellum. **(A)** OXPHOS complex assembly in B05 (lanes 1–3) and wild type (lanes 4–6) cerebellum by Western blot using an assembly-specific OXPHOS antibody cocktail. **(B)** Quantification of western blot data in (B). CI, CII, CIII and CV bands are each normalized to CIV. Data is in triplicate. Error bars show means ± SEM. T-test, * P < 0.05. **(C)** Mitochondrial respiration analysis of B05 and wild type cerebellum in the presence of ADP or rotenone. Data is shown as the percent maximal respiration which was calculated in the presence of TMPD/Ascorbate. Samples were run in triplicate. Error bars show means ± SEM. T-test, * P< 0.05.

Next, we analyzed OXPHOS complex protein assembly by western blotting using an assembly specific antibody cocktail (Fig 1A). 20-week old B05 and wild type cerebellar lysates were prepared in SDS sample buffer, but were not boiled to preserve complex assembly, as per manufacturer’s instructions. As a result, we did not include a typical loading control but instead relied on the reproducibility of the complex IV subunit across samples as our normalization factor. Compared to complex IV which showed no change in assembly, the complex I subunit levels were significantly reduced indicating decreased complex I assembly (Fig 1B). No significant reduction was seen in complex II, III or V (ATPase).

Finally, we examined respiratory activity of adult B05 cerebellum compared to wildtype cerebellum using permeabilized whole tissue respirometry (Fig 1C). The benefit of whole tissue versus mitochondrial preparations is to prevent bias by including unhealthy or damaged mitochondria in the analysis. Our results show a significant reduction of complex I-driven respiration (Glu+Mal+ADP) in B05 cerebellum compared to wild type cerebellum suggestive of a functional deficit in complex I (Fig 1C). To confirm this, the complex I inhibitor, rotenone, was applied (Glu + Mal + ADP + Rotenone) and the difference in total respiration between B05 and wild type was abolished, further suggesting diminished complex I activity in SCA1 (Fig 1C). Taken together, the above transcriptional, biochemical and physiological data indicate complex I dysfunction occurs during SCA1.

### Ad libitum succinic acid as a treatment strategy for B05 mice

**Fig 2:**
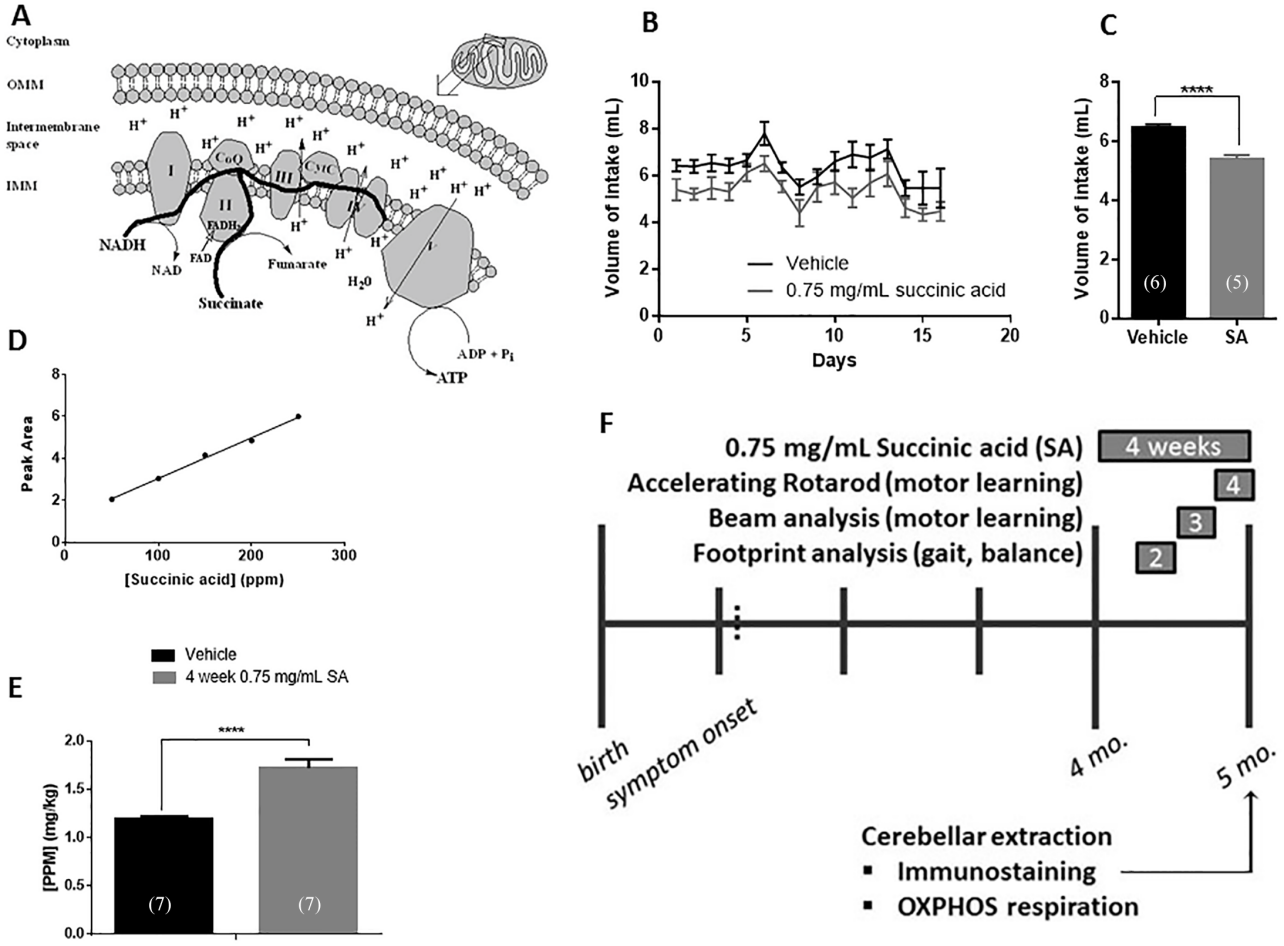
SA as a treatment strategy for B05 mice. **(A)** Schematic (drawn in ChemDraw) showing mitochondrial inner membrane (IMM) OXPHOS complexes, flow of protons (H^+^), redox and phosphorylation reactions, and the flow of electrons (thick black lines). OMM = outer mitochondrial membrane. **(B)** Volume of water measured daily in untreated and succinic acid treated mice. **(C)** Average water intake over a two-week period in treated (SA) (mean = 5.415 ± 1.069 mL) and untreated (6.463 ± 1.058 mL) mice. Error bars represent means ± SEM. T-test, **** P < 0.0001. **(D)** Standard curve of succinic acid by HPLC. Slope = 0.01939 ± 0.0007448, R^2^ = 0.9956. **(E)** succinic acid concentrations in cerebellar tissue detected in untreated and 4-week treated wild type mice. Error bars represent means ± SEM. T-test, P < 0.0001. **(F)** Schema of treatment plan. Symptom onset begins approximately 5 weeks after birth. We began treatment of 0.75 mg/mL succinic acid beginning at 4 months of age and continued for four weeks. Subjects were behaviorally tested during weeks 2 (footprint analysis), 3 (beam analysis) and 4 (accelerating rotarod). Following treatment, 5 month-old mice were sacrificed and cerebellar tissue was harvested for oxygen respiration analysis and neuropathology.

As a strategy to overcome potential OXPHOS complex I dysfunction, we hypothesized that bypassing complex I in the electron-transfer chain may have therapeutic potential. Succinic acid, a Krebs cycle-generated metabolite, donates electrons directly into complex II and thus could be used to bypass complex I [25–27, 30] (Fig 2A). Additionally, succinic acid is inexpensive, endogenous and FDA-approved making it an attractive therapeutic [43–47]. A concentration of 0.75 mg/mL of succinic acid dissolved in the cage drinking water and administered ad libitum was chosen based on preliminary dose-response data. We first tested whether mice will drink 0.75 mg/mL succinic acid by weighing water bottles of treated and untreated mice daily for 16 days (Fig 2B). Volume of intake did not change drastically over the 16-day period. Minor variability was observed in both treatment groups likely due to the timing of the measurement on that day. Overall, there was a significant decrease of about 1 mL per day in the intake volume of treated versus untreated water (Fig 2C). The intake volume per treated mouse was consistent however, resulting in an intake dosage of approximately 4 mg of succinic acid per mouse per day.

We next sought to determine if *ad libitum* succinic acid dissolved in the cage drinking water would bypass the blood brain barrier. To address this, we adapted a protocol for measuring succinic acid concentrations in olive fruit strains to our cerebellar samples [47]. A biological vehicle was spiked with known succinic acid concentrations and used to develop a standard curve by HPLC (Fig 2D). PBS-washed cerebellar tissue from vehicle-treated and succinic acid-treated mice were detected by the HPLC assay. Succinic acid-treated cerebellar tissue featured approximately 133% more succinic acid than endogenous levels in the untreated cerebellar tissue (Fig 2E).

Having established that succinic acid can be effectively administered via cage water bottles and that it crosses the blood brain barrier, we designed the following protocol scheme to administer succinic acid to B05 mice (Fig 2F). B05 mice first exhibit neuropathological and behavioral deficits at 12 weeks of age [24]. In order to allow these deficits to develop, we began treatment at 4 months of age continuously for four weeks. During week 2 of treatment, animals were analyzed for gait and balance via a footprint assay; during week 3 of treatment, animals were analyzed for motor learning via balance beam analysis; and during the final week of treatment, animals were analyzed for balance and motor learning via the accelerating rotarod paradigm. Following treatment, at 5 months of age, mice were sacrificed and their cerebella were extracted for immunostaining and mitochondrial respiration.

### Succinic acid treatment augments OXPHOX complex function in B05 mice cerebellum

**Fig 3:**
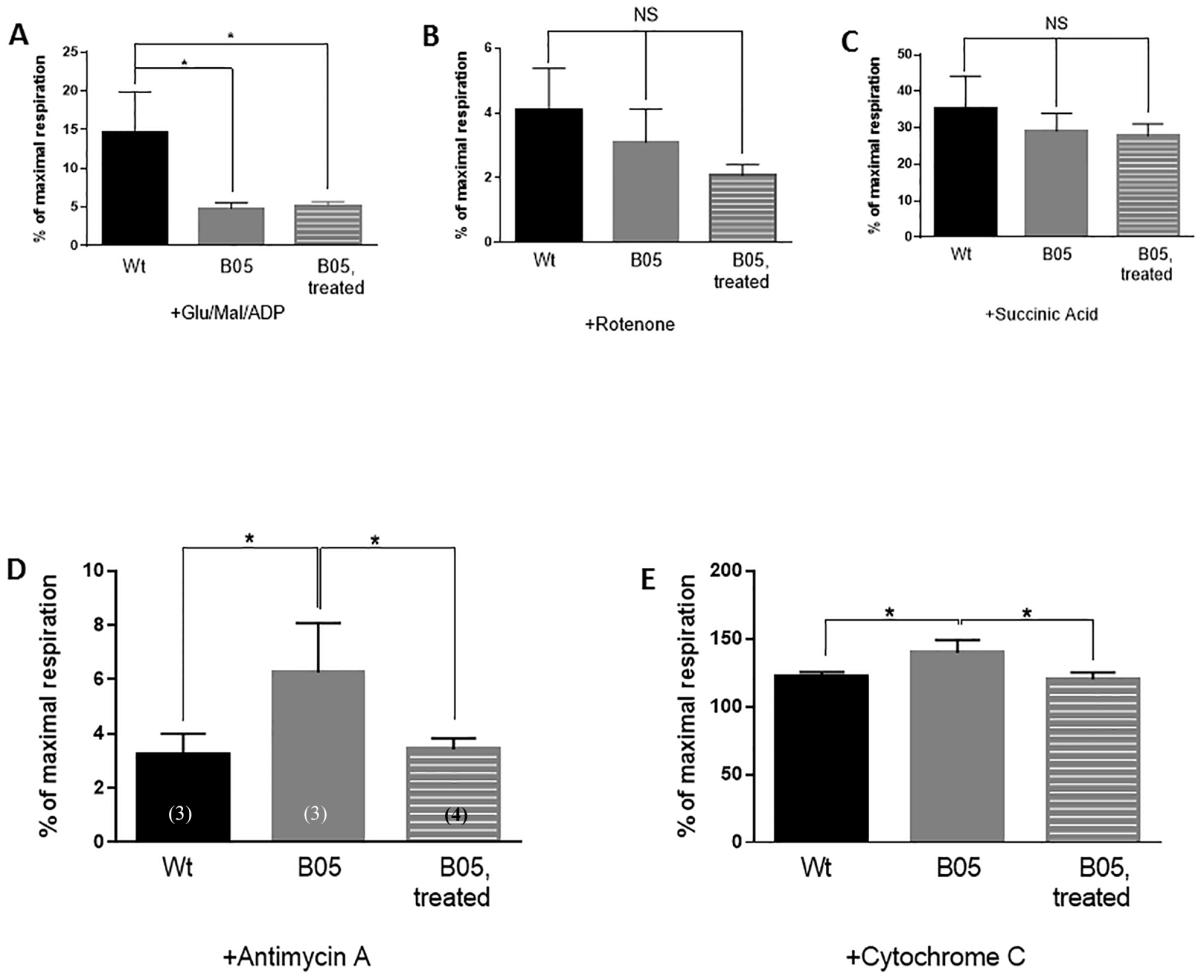
1 month SA treatment restores OXPHOS complex function in adult B05 mice. **(A-E)** Oxygen respiration response of wild type untreated, B05 untreated and B05 treated mice cerebellar tissue to the addition of **(A)** 1 mM ADP, **(B)** 0.5 μM rotenone, **(C)** 10 mM succinic acid, **(D)** 5 μM antimycin A, and **(E)** 10 μM cytochrome C. Responses are the percent of maximal respiration as measured by the oxygen respiration response to the simultaneous addition of 0.5 mM TMPD and 2 mM ascorbate. Error bars represent means ± SEM. One-way ANOVA with Tukey’s post-hoc test, * P < 0.05, NS = non-significant.

The effects of succinic acid treatment on oxygen consumption was assessed by respirometry. The significant reduction in complex I-driven respiration in the B05 cerebellum compared to wild type cerebellum (Fig 1C) persisted in succinic acid-treated B05 cerebellar tissue (Fig 3A and 3B). Furthermore, inhibition of complex I with rotenone had no effect on oxygen consumption (Fig 3B). With selective activation of complex II-driven respiration (Glu+Mal, ADP, Rotenone, and succinic acid), no significant differences were found between groups (Fig 3C), suggesting along with the protein assembly data (Fig 1A and 1B), that complex II and further downstream complexes, appear to be relatively intact. Moreover, succinic acid treatment did not disrupt complex II or downstream oxygen consumption. However, interrogation of complex III activity with the Qi site-specific complex III-inhibitor antimycin A [48] shows that B05 cerebellar mitochondria were less affected by complex III blockade (Fig 3D). Importantly, this effect is rectified with succinic acid treatment (Fig 3D). Finally, exploration of mitochondrial membrane integrity revealed that B05 cerebellar mitochondria responded significantly to cytochrome C addition (Fig 3E). This finding is suggestive of either low cytochrome C pools within B05 cerebellar tissue, or that SCA1 pathogenesis is initiating damage to the mitochondrial membrane [34]. Both potential causes will require further investigation. Importantly, the effect of any damage is reversed with succinic acid treatment (Fig 3E).

### Succinic acid treatment ameliorates molecular layer and Purkinje cell layer degeneration in B05 mice cerebellum

**Fig 4:**
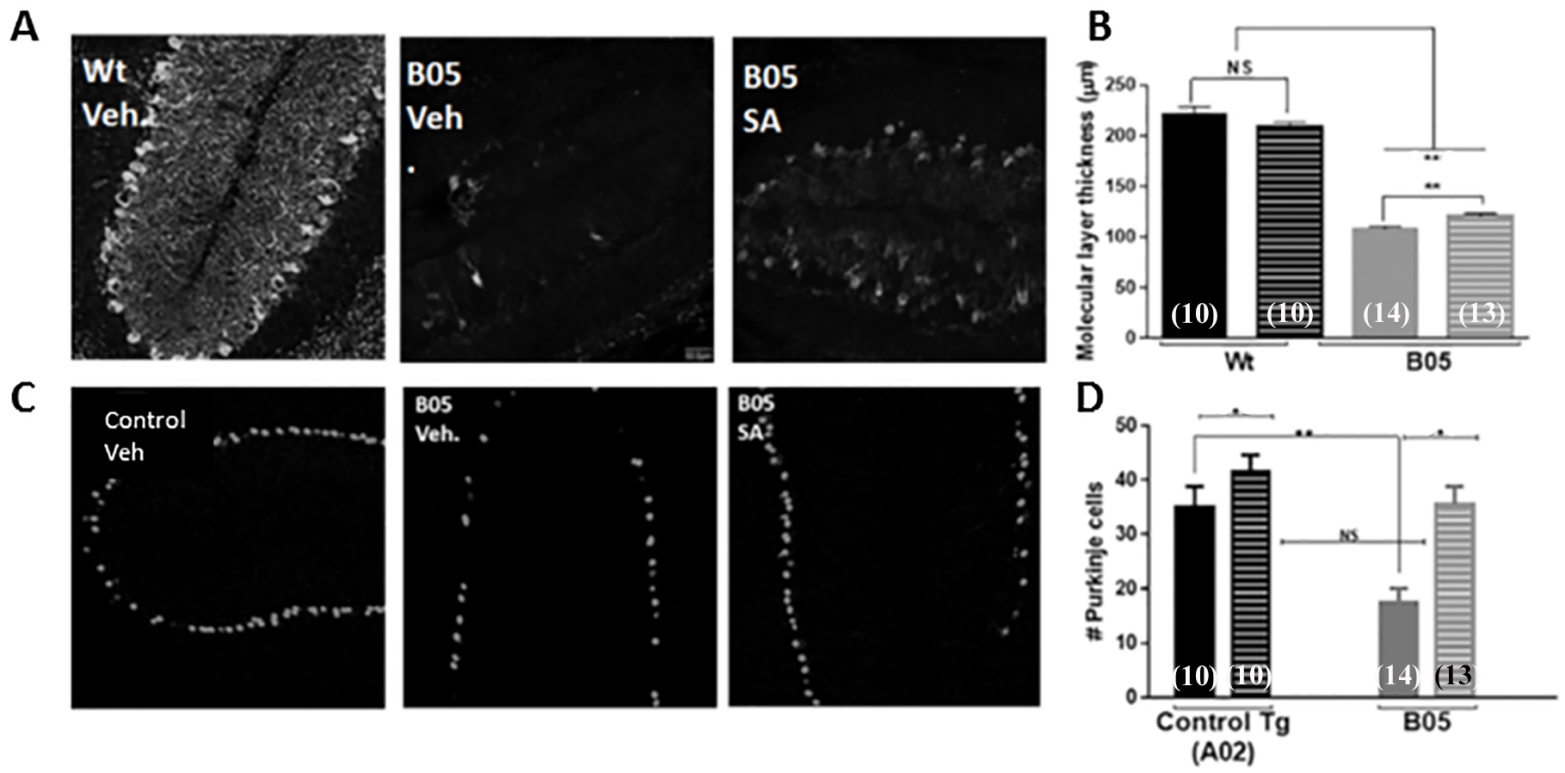
1 month SA treatment slows Purkinje neuron degeneration in adult B05 mice. **(A)** Representative calbindin-stained cerebellar primary fissure images from untreated wild type, untreated B05 and treated B05 mice. **(B)** Molecular layer thickness, a measure of calbindin-positive Purkinje dendritic length, in treated and untreated wild type and B05 cerebellum. **(C)** Representative ATXN1-stained cerebellar primary fissure images from untreated control transgenic (A02), untreated B05 and treated B05 mice. **(D)** Purkinje nuclei counts in treated and untreated A02 and B05 cerebellum. Error bars represent means ± SEM. Two-way ANOVA,* P < 0.05, ** P < 0.01.

Fixed, frozen sagittal sections of cerebellar tissue were cut to 50 μm thickness and stained for calbindin, a marker of Purkinje cell soma and dendrites or 11NQ, an ataxin-1 antibody which preferentially stains Purkinje cell nuclei in SCA1 transgenic mouse models. Images of sagittal sections surrounding the primary fissure were captured by confocal microscopy. Purkinje cell dendritic length was measured in calbindin-stained tissue from the soma to its endpoint towards the primary fissure. Representative images for untreated (vehicle-treated) wild type, untreated B05 and treated B05 are shown in Fig 4A. Average molecular layer thickness, a measure of Purkinje cell dendrite extension, is graphed in Fig 4B. No difference is seen in treated or untreated wild type. A significant reduction in molecular layer length is seen in untreated B05. And a significant increase in molecular layer length is detected with *ad libitum* treatment of succinic acid (Fig 4B).

Purkinje cell nuclei were counted in 11NQ-stained slices from an untreated transgenic control mouse (A02, which expresses ATXN1[30Q] diffusely in Purkinje cell nuclei but does not exhibit SCA1 neuropathological or behavioral deficits), untreated B05 (which expresses aggregated ATXN1[82Q] in Purkinje cell nuclei) and treated B05. Representative images are shown in Fig 4C, and average number of Purkinje nuclei are graphed in Fig 4D. There was no statistical difference in the number of 11NQ-positive Purkinje cells between treated and untreated wild type cerebellum (Fig 4D). As with the molecular layer thickness measure, Purkinje cell nuclei were significantly reduced in untreated B05 but that reduction was prevented with *ad libitum* treatment of succinic acid (Fig 4C and 4D).

### Succinic acid treatment improves the cerebellar ataxia phenotype in B05 mice

**Fig 5:**
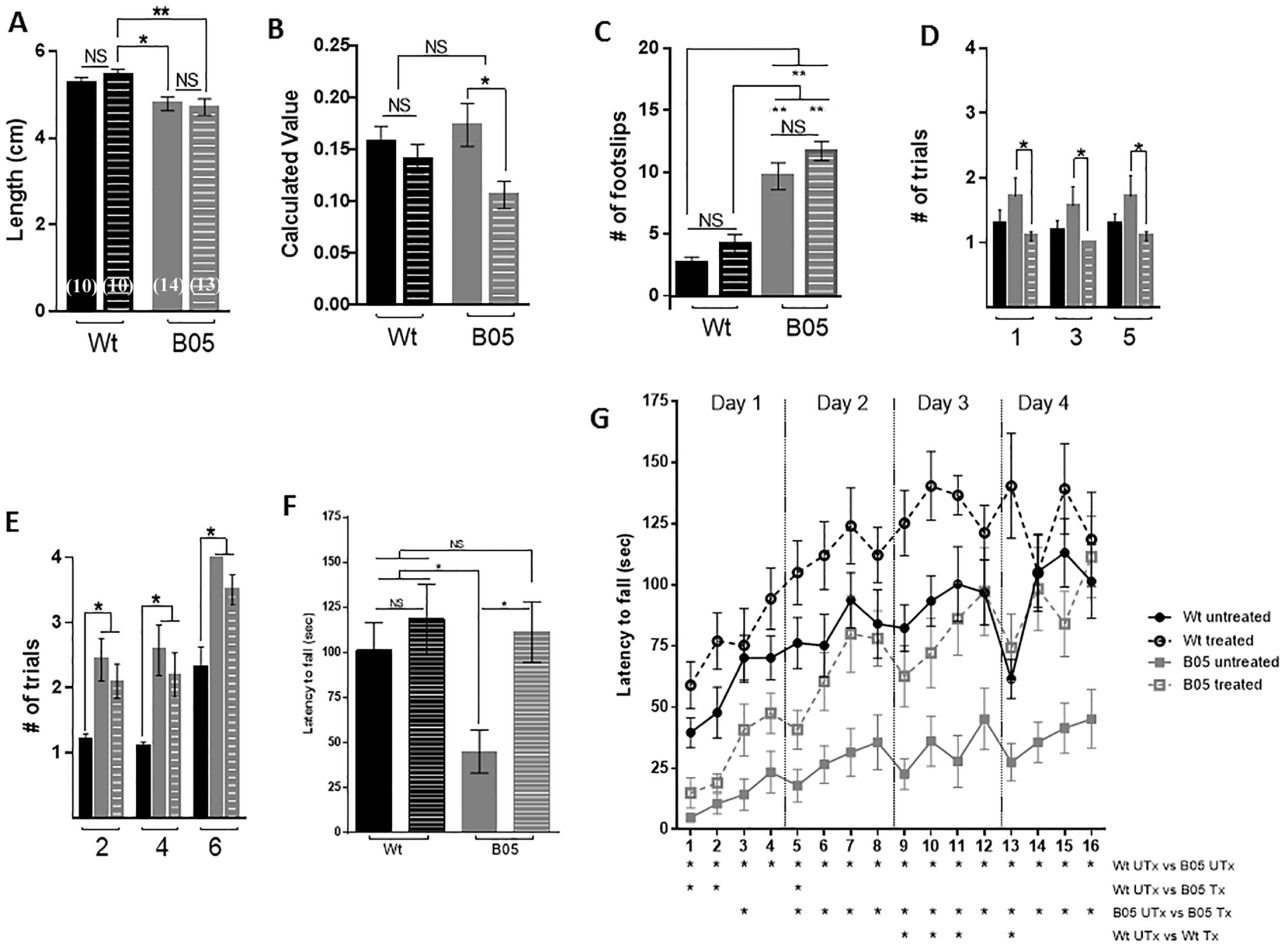
1 month SA treatment improves cerebellar behavior in adult B05 mice. **(A)** Step-length, measured by the footprint runway assay during week 2 of treatment, in wild type (black bars) and B05 (gray bars) untreated (solid bars) and treated (dashed bars) mice. **(B)** Alternation coefficient, measured by the footprint runway assay during week 2 of treatment. Calculated values reflect the extent of shuffle gait. **(C)** Number of hind left foot slips during successful crossing of beam 3 on testing day during week three of treatment. **(D)** Number of trials needed for a successful cross of square beams 1, 3 and 5 on testing day during week 3 of treatment. **(E)** Number of trials needed for a successful cross of round beams 2, 4 and 6 on testing day during week 3 of treatment. **(F)** Latency to fall during trial 16 of the accelerating rotarod test during week 4 of treatment. **(G)** Latency to fall during all trials of the accelerating rotarod test during week 4 of treatment. Error bars represent means ± SEM. Two-way ANOVA, * P < 0.05, ** P < 0.01.

Gait was measured during the second week of the four-week ad libitum treatment of succinic acid using the footprint assay. The footprint assay is a simple paradigm in which hind feet are painted with blue (left foot) or red (right foot) paint and mice are allowed to walk on a paper-lined runway towards a goal box. Each mouse performed the assay successfully one time. Gait width, step length, linear movement and alternation coefficient was analyzed [33, 35]. We did not detect a difference in gait width or linear movement due to genotype or treatment condition. The lack of significant results is likely due to a combination of the insensitivity of the assay and the reduced time of treatment. When calculating step length, the untreated wild type mouse did not show a significant increase in step length compared to the untreated or treated B05 mice. However, both the untreated and treated B05 mouse showed significantly reduced step length compared to the treated wild type mouse (Fig 5A). Alternation co-efficient, a measure of shuffle, was not affected in wild type mice by treatment. A trend of increased shuffle was seen between the untreated B05 mouse and wild type mice, although the increase was not significant. However, a statistically significant reduction in shuffle was found in B05 mice due to treatment (p < 0.05) (Fig 5B).

Footslips and beam cross attempts were assessed during the third week of treatment via the balance beam test [33, 35] (Fig 5C–5E). B05 mice made a significantly greater number of footslips on beam 3 than wild type mice, and succinic acid treatment did not alter the number of footslips (Fig 5C). Fig 5D shows the number of attempts made to successfully cross the square beams (beams 1, 3 and 5). A trend towards an increased number of attempts is seen in untreated B05 compared to untreated wild type mice. However, we found a significant decrease in the number of attempts to cross each of the three square beams by treated B05 mice compared to untreated B05 mice. (Fig 5D). Fig 5E shows the number of attempts made to successfully cross the round beams (beams 2, 4 and 6). A significant increase in the number of attempts is seen in untreated B05 compared to untreated wild type (Fig 5E). Unlike with the square beams, succinic acid treatment did not decrease the number of attempts that B05 mice took to successfully cross the round beams (Fig 5E). The balance beam data as a whole suggests that succinic acid treatment may improve cerebellar motor behavior in the SCA1 B05 mouse.

Improvement to the ataxia phenotype was directly assessed during the fourth week of treatment via the accelerated rotarod. Fig 5F shows the data from the final trial and Fig 5G shows the results of all 16 trials over four days for untreated and treated wild type mice, and untreated and treated B05 mice. Untreated wild type mice showed gradual improvement over the sixteen trials, and improved their latency-to-fall time by one minute at trial 16 (Fig 5F and 5G). Surprisingly, treated wild type mice performed even better than untreated wild type mice showing a significant improvement from untreated mice during trials 9, 10, 11 and 13, but not the final trial (Fig 5F and 5G). In contrast, untreated B05 mice were unable to remain on the rotarod for more than a couple of seconds on trial 1. While they also showed improvement over the 16 trials, their performance during the final trial remained under 50 seconds (Fig 5F and 5G). They performed significantly worse than untreated wild type mice during each of the 16 trials. Treated B05 mice performed equally to the untreated B05 mice in early trials, yet showed significant improvement, when compared to untreated B05 controls, in trials other than the first, second and fourth. Furthermore, by the final trial, treated B05 mice performed equally to wild type mice (Fig 5F).

Our behavioral tests, administered during the final three weeks of the four-week treatment period, show that succinic acid improves cerebellar behavior in B05 mice. Improvement in shuffle, but not in step length, was seen during week 2 of treatment. Improvement in performance on the tested beams (square) were seen during week 3 of treatment. No improvement in footslips was shown during week 3 of treatment. During week 4 of treatment, latency-to-fall during the accelerated rotarod training paradigm improved starkly in treated B05 mice, which began trial 1 at the same level as dysfunction as the untreated mice. In concert, our behavioral data strongly favors a model by which succinic acid *ad libitum* treatment ameliorates behavioral decline in B05 mice caused by Purkinje cell degeneration.

### Chronic succinic acid treatment in adult mice does not perturb overall metabolism

**Fig 6:**
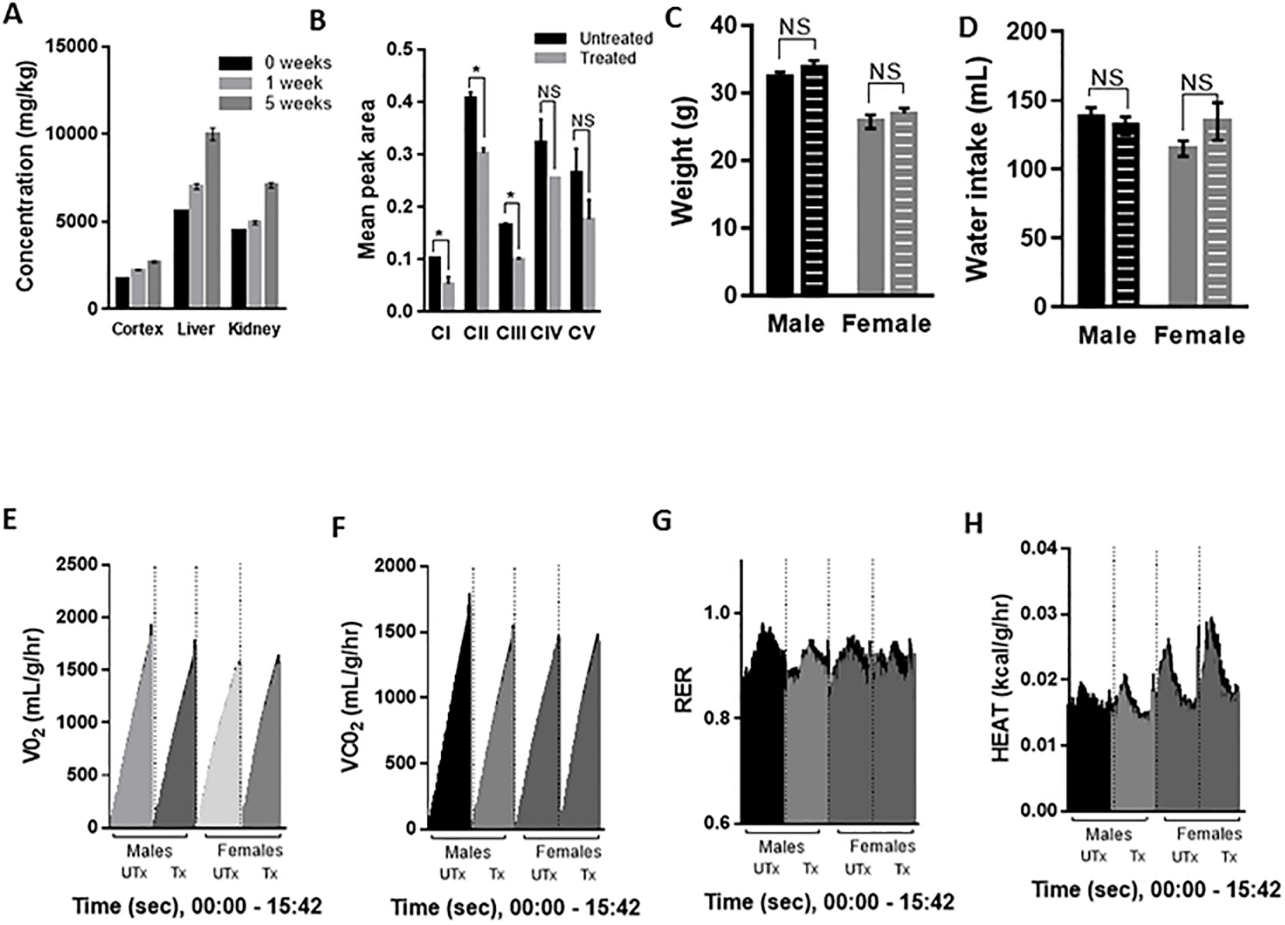
Chronic (> 4 months) SA treatment of adult mice does not alter overall metabolism. **(A)** HPLC analysis of cortex, liver and kidney from wild type mice treated with 0.75 mg/mL succinic acid for 0, 1 or 5 weeks shows elevated levels in these regions. Error bars represent means ± SEM. **(B)** Chronic treatment of adult mice does alter complex I, complex II and complex III assembly in cerebellar tissue of wild type mice as determined by western blotting with an OXPHOS complex assembly-dependent antibody cocktail. Error bars represent means ± SEM. * P < 0.05. **(C)** Weight of chronically treated male and female wild type mice is not significantly different from untreated. Error bars represent means ± SEM. NS = non-significant. **(D)** Water intake during running wheel assay of chronically treated male and female wild type mice is not significantly different from untreated. Error bars represent means ± SEM. NS = non-significant. **(E-H)** Metabolic cage testing of chronically treated male and female wild type mice and untreated male and female wild type mice showing **(E)** oxygen consumption (VO_2_), **(F)** carbon dioxide production (VCO_2_), **(G)** respiratory exchange ratio (RER) and **(H)** energy expenditure (HEAT). X-axes shows the time scale from 0:00 to 15:42 (hr:min) of recorded time. Black points indicate SEM. Statistical analyses are described in the results.

Given the ubiquitous nature of succinic acid in the body and the impact that increasing succinic acid levels can have on metabolism and physiology [49–54], we further tested the effects of *ad libitum* succinic acid treatment in adult wild type mice apart from its function on Purkinje cells. First, we measured the concentration of succinic acid delivered to the cortex, liver and kidneys in response to cage water treatment for 1 week or 5 weeks (Fig 6A). A dose response was found in each of the three regions, with the highest concentrations of succinic acid found in the liver (Fig 6A). Next, we turned to a chronic (≥ 4 months) *ad libitum* treatment model to investigate potential side effects of treatment. We analyzed cerebellar OXPHOS complex assembly and detected reduced assembly of complexes I, II and III in chronically treated wild type mice. Complex IV and V assembly were not affected (Fig 6B). Next, we measured the weight of adult wild type chronically treated and untreated mice. No significant difference in weight of adult males or females due to chronic treatment was found (Fig 6C). Average water intake over a 24-hour period was not different between chronically treated and untreated mice (Fig 6D).

To measure potential changes to overall metabolism due to chronic treatment, chronically treated and untreated A02 mice were placed into metabolic cages with free access to food and either treated or untreated water. Mice were allowed 8 hours of acclimation time before beginning the testing period in 12-hour dark and 12-hour light cycles. Four variables were recorded 3–4 times per hour over a 15.75-hour period that included 7 hours of dark time and 8.45 hours of light: oxygen consumption (VO_2_), carbon dioxide production (VCO_2_), respiratory exchange ratio (RER) and energy expenditure (HEAT) (Fig 6E–6H). VO_2_ (Fig 6E), VCO_2_ (Fig 6F), RER (Fig 6I) or HEAT (Fig 6H) in untreated and treated male and female mice are shown over time. Repeated measures two-way ANOVA was used to determine the effects of treatment and sex on the above variables. No statistical difference due to sex or treatment was found in VO_2_, VCO_2_ or RER. A main effect (p < 0.001) was found in energy expenditure. Tukey’s post-hoc analysis revealed an interaction of sex x time among treated subjects (p< 0.05); Fig 6H shows increased energy expenditure measures in female treated mice compared to male treated mice. Further analysis of the energy expenditure results was investigated with multiple comparisons t-test analysis to determine the effects of treatment or sex at each time point. A single time point (at 3 hrs:54 mins, dark cycle) showed a statistically significant effect of treatment on energy expenditure in female mice (P < 0.05). A sex effect on energy expenditure was found in the untreated subjects that persisted from 1 hr:48 mins to 5hrs:6 mins (first 20 minutes in light, remaining 3 hours in dark) (P < 0.05 or lower at each time point). A more pronounced sex effect on energy expenditure was found in the treated males versus females beginning at 1 hr:48 mins and occurred discontinuously through the remainder of the recorded period (first 20 minutes in light, followed by 12 hours in dark, followed by 40 minutes in light) (P < 0.05 or lower).

To further test the metabolic effects of chronic succinic acid treatment, we tested circadian free-running activity. Succinate treatment had no significant effect on the free-running circadian period in DD or the time of peak activity 12:12LD (Table 2) indicating that the circadian timing of activity and the period of the circadian pacemaker driving the activity rhythm in DD are not changed by the treatment. None of the measures of mean activity level in 12:12LD or DD were significantly altered by succinate treatment (see Table 2). There were no significant interactions between succinate treatment and sex effects. Yet, mice treated with succinic acid are consistently less active for all measures of activity (Table 2). Further analysis will be needed to assess the effect of chronic treatment on Purkinje cells and cerebellar behavior.

**Table 2:** Chronic (> 4 months) SA treatment of adult mice does not alter overall activity.

Variable	Control	Treated
XLD	30.4 ± 3.2	26.2 ± 5.0
XL	14.8 ± 2.2	11.6 ± 2.8
XD	46.1 ± 6.0	40.8 ± 8.3
XDD	35.3 ± 4.2	24.6 ± 5.0
PHLD	23.4 ± 0.5	22.7 ± 0.6
TAUDD	23.65 ± 0.07	23.48 ± 0.11
Age	300 ± 15	297 ± 14

Data table shows mean +/-SEM activity levels (average wheel revolutions per ten minutes), circadian timing of activity (hours) in LD and DD, and age (days). XLD = mean number of wheel turns in 12:12 LD per 10 minutes; XL = mean number of wheel turns in L during LD (per 10 minutes); xd = mean number of wheel turns in D during LD; XDD = mean number of wheel turns during DD (per 10 minutes); TAUDD = period of the circadian wheel-running rhythm during DD (in hours); PHLD = time of peak activity during LD. None of the variables were significantly different between control and treated mice.

## Conclusions

Deficits in complex I of the mitochondrial electron transport chain, in particular, have reported to affect and/or accompany neurodegeneration and toxicity in vulnerable neurons [55, 56]. A decrease of complex I activity occurs with age, and is further pronounced in the striatal and nigral neurons of Parkinson’s disease [21, 22]. Substantial down-regulation of complex I subunit genes are found in Alzheimer’s disease [20]. Loss of frataxin function in Friedrich’s ataxia results in accumulation of mitochondrial iron that selectively reduces complex I activity [14, 19]. Additionally, several mutations in the nuclear-encoded subunits of complex I are reported to cause Leigh syndrome [14, 15]. In the case of Friedrich’s ataxia and Leigh syndrome, both juvenile fatal disorders, complex I deficiency disrupts cerebellar function as is evident by the common symptom of cerebellar ataxia [14, 15, 19]. Most recently, complex I deficiency and other mitochondrial dysfunction was reported in a knock-in ATXN1[154Q] mouse model [23].

Our present study investigates complex I function in the cerebellum of the B05 mouse which selectively expresses the ATXN1[82Q] transgene. Advantages of this model include the Purkinje neuron- specificity of transgene expression and the biological relevance of the 82Q-length expanded polyglutamine tract [57, 58]. The impact of ubiquitous succinic acid delivery specifically on cerebellar cells exposed to ATXN1[82Q] in our mouse model can be examined.

We found that succinic acid boosts cerebellar function in the B05 mouse following short-term treatment. Succinic acid crosses the blood brain barrier and penetrates cerebellar tissue. In whole cerebellar tissue, succinic acid bypasses dysfunctional complex I without disrupting complex II or downstream oxygen consumption, restores complex III inhibition by antimycin A, and prevents damage of outer mitochondrial membranes. Within Purkinje neurons, succinic acid diminishes Purkinje dendritic atrophy and averts loss of Purkinje soma. On a behavioral level, succinic acid lessens the cerebellar ataxia phenotype.

Succinic acid is a low cost, readily available organic compound that has been reported to bypass complex I and stimulate activity of complex II [25–27]. Inhibition of succinic acid by the reversible inhibitor malonate and the irreversible inhibitor nitropropionic acid causes neurodegeneration in animal models [59–61]. In our mice model, it is an effective means of circumventing complex I dysfunction and ameliorating the effects of ATXN1[82Q]-driven neurodegeneration in B05 Purkinje cells.
